# Supplementary figures and images for: Modeling the consequences of age-linked rDNA hypermethylation with dCas9-directed DNA methylation in human cells
Source: PLoS One. 2024 Dec 12;19(12):e0310626. doi: 10.1371/journal.pone.0310626 (PMC11637357; doi:10.1371/journal.pone.0310626)

Supplementary Figure 2. relevant features of the human rDNA repeat (Genbank GL000220.1)

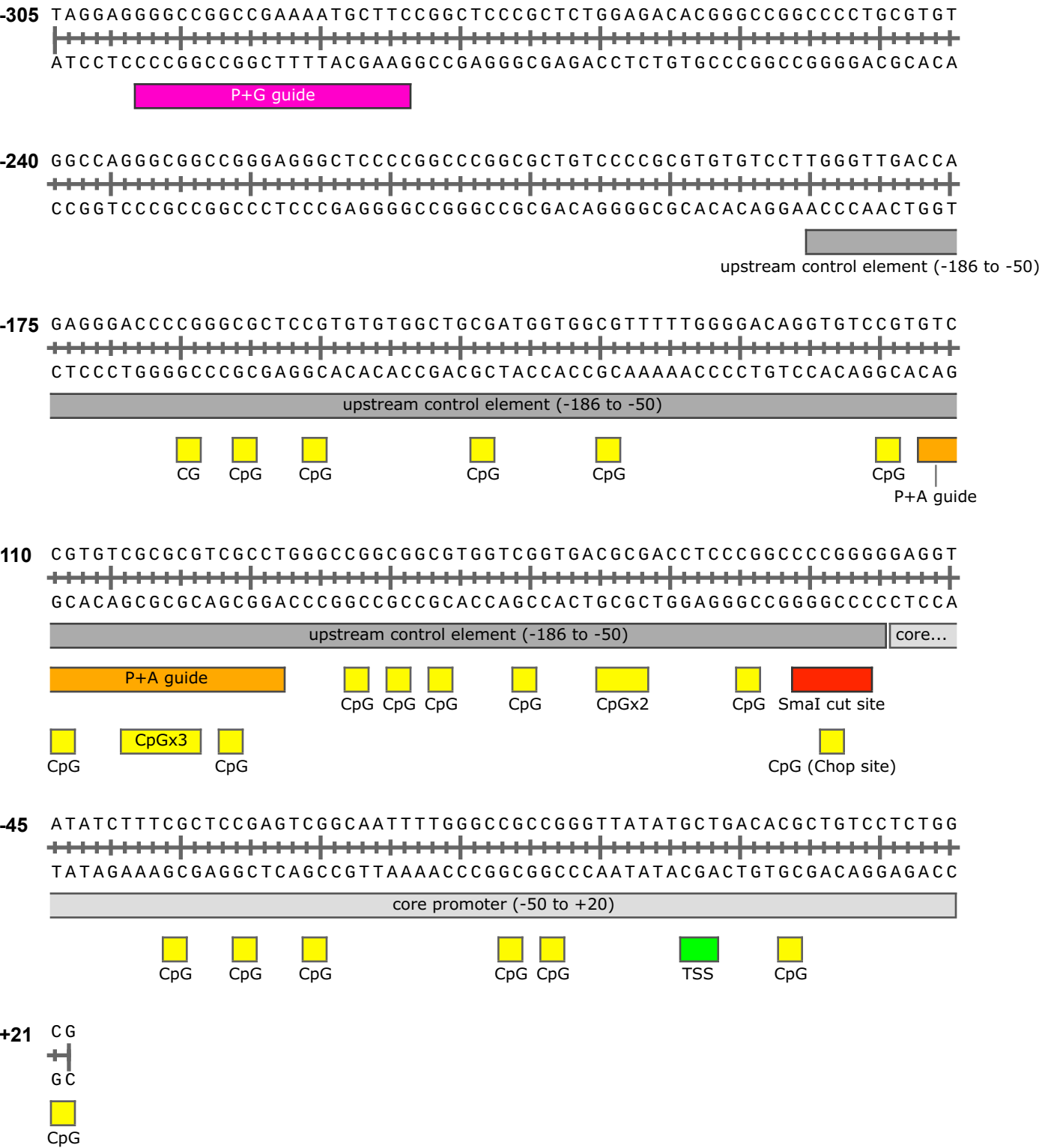

Supplement: S2 Fig — (PDF) [file pone.0310626.s002.pdf]
