# Supplementary material for: Modeling the consequences of age-linked rDNA hypermethylation with dCas9-directed DNA methylation in human cells
Source: PLoS One. 2024 Dec 12;19(12):e0310626. doi: 10.1371/journal.pone.0310626 (PMC11637357; doi:10.1371/journal.pone.0310626)
Supplement: S1 Table — (PDF) [file pone.0310626.s005.pdf]

Supplementary Table I. Primers Used.

Name	Sequence	Use
dCas9-Dnmt3a3l-fwd	tcctaccctcgtaaaggtaccatggacaagaagtattcaatc	cloning pXlone-dCas9-DNMT3A3L
dCas9-Dnmt3a3l-rev	ctcgcaggggaggtggtctactagtttacttgtacagctcgtc	cloning pXlone-dCas9-DNMT3A3L
Gibson_puro-2-Fwd	aactaaacccatatggccaccatgaccgagtacaagcccacgg	cloning pKH011-puro
Gibson_puro_Rev	tcg agg ctg atc agc gag ctc tag atc agg cac cgg gct tgc g	cloning pKH011-puro
Guide_Promoter+_A_Fwd	CACCGTCCGTGTCGCGCGTCGCCT	Referred to as P+A sgRNA
Guide_Promoter+_A_Rev	AAACAGGCGACGCGCGACACGGAC	Referred to as P+A sgRNA
Guide_28S+_B_Fwd	CACCCGTTAAGAGGTAAACGGGTG	Referred to as 28SB sgRNA
Guide_28S+_B_Rev	AAACCACCCGTTTACCTCTTAACG	Referred to as 28SB sgRNA
NTC_sgRNA1_F	CACCGGATACGGTCGCGTGTTAC	Referred to as NTC sgRNA
NTC_sgRNA1_R	AAACGTAACACGCGACCGTATCC	Referred to as NTC sgRNA
Safe_sgRNA1_F	CACCGGATTCTTCTGACTCATT	Referred to as Safe sgRNA
Safe_sgRNA1_R	AAACAATGAGTCAGAAGAATCC	Referred to as Safe sgRNA
P+G-oligo_F	CACCGGGCCGGCCGAAAATGCTTC	Referred to as P+G sgRNA
P+G-oligo_R	AAACGAAGCATTTTCGGCCGGCCC	Referred to as P+G sgRNA
rDNA2F	AACGTGAGCTGGGTTTAG	ddPCR
rDNA2R	CTCGTACTGAGCAGGATTAC	ddPCR
rDNA2probe	/5HEX/TGGCAACAA/ZEN/CACATCATCAGT/3IABkFQ/	ddPCR
Chrom11F	AGCCTTTACTACAGAACATCTCAC	ddPCR
Chrom11R	TCTTTCCTCTCCCTTCCCTTTA	ddPCR
Chrom11probe	/56-FAM/ACTCCAACA/ZEN/AATGCTTGCTGACGC/3IABKFQ/	ddPCR
Promoter Cut Site Fwd	TGCGATGGTGGCGTTTTTG	methylation-sensitive qPCR
Promoter Cut Site Rev	CCGACTCGGAGCGAAAGATA	methylation-sensitive qPCR
Promoter no cut site Fwd	CGATCCTTTCTGGCGAGTCC	methylation-sensitive qPCR
Promoter no cut site Rev	GACACACGAGGGACCGAAG	methylation-sensitive qPCR
5' ETS Cut Site Fwd	TCT AGC GAT CTG AGA GGC GT	methylation-sensitive qPCR
5' ETS Cut Site Rev	CAG CGC TAC CAT AAC GGA GG	methylation-sensitive qPCR
5'ETS no cut site Fwd	GGT GTT TCC TCG TAC CGC A	methylation-sensitive qPCR
5'ETS no cut site Rev	AAG GCT TTT CTC ACC GAG GG	methylation-sensitive qPCR
18S Cut Site Fwd	GAT GGT AGT CGC CGT GCC	methylation-sensitive qPCR
18S Cut Site Rev	GCC TGC TGC CTT CCT TGG	methylation-sensitive qPCR
18S no cut site Fwd	GGCCCTGTAATTGGAATGAG	methylation-sensitive qPCR
18S no cut site Rev	GCTCCCAAGATCCAACTACG	methylation-sensitive qPCR
5.8S Cut Site Fwd	ACTCGGCTCGTGCGTC	methylation-sensitive qPCR
5.8S Cut Site Rev	GCGACGCTCAGACAGG	methylation-sensitive qPCR
5.8S no cut site Fwd	CTTAGCGGTGGATCACTCGG	methylation-sensitive qPCR
5.8S no cut site Rev	TGCGTTCGAAGTGTCGATGA	methylation-sensitive qPCR
28S Cut Site Fwd	AGAGGTAAACGGGTGGGGTC	methylation-sensitive qPCR
28S Cut Site Rev	GGGGTCGGGAGGAACGG	methylation-sensitive qPCR
28S no cut site Fwd	aac gtg agc tgg gtt tag	methylation-sensitive qPCR
28S no cut site Rev	ctc gta ctg agc agg att ac	methylation-sensitive qPCR
45S pre-rRNA Fwd	GAACGGTGGTGTGTCGTT	RT-qPCR
45S pre-rRNA Rev	GCGTCTCGTCTCGTCTCACT	RT-qPCR
28S rRNA Fwd	AGAGGTAAACGGGTGGGGTC	RT-qPCR

28S rRNA Rev	GGGGTCGGGAGGAACGG	RT-qPCR
5S rDNA Fwd	GCCATACCACCCTGAACG	ChIP-qPCR
5S rDNA Rev	AGCCTACAGCACCCGGTATT	ChIP-qPCR
Gapdh_Fwd	ACCACAGTCCATGCCATCAC	RT-qPCR
Gapdh_Rev	GGATGATGTTCTGGAGAGCCC	RT-qPCR
hrDNA bis-P Fwd	GTTTTTGGGTTGATTAGAGGGATTT	BSAS
hrDNA bis-P Rev	ACAAACAAAACTATCTACC	BSAS
