# Supplementary material for: Modeling the consequences of age-linked rDNA hypermethylation with dCas9-directed DNA methylation in human cells
Source: PLoS One. 2024 Dec 12;19(12):e0310626. doi: 10.1371/journal.pone.0310626 (PMC11637357; doi:10.1371/journal.pone.0310626)
Supplement: Attachment — Submitted filename: 141154_1_rebuttal_3031276_sdzxpv.pdf [file pone.0310626.s007.pdf]

Full Revision

Manuscript number: RC-2023-02221
Corresponding author(s): Abigail Buchwalter

1. General Statements

We would like to thank the reviewers for their thoughtful evaluation of our work. Our point-by-point responses to reviewer critiques follow below. Please note that any referenced changes to the manuscript are highlighted in yellow in the revised manuscript text.

2. Point-by-point description of the revisions

Response to Common Critiques

1. Reviewers 1 and 2 state that some elements of this study confirm previously published results (many in murine systems). However, the reviewers also acknowledge that the mouse and human rDNA repeats may be subject to quite distinct regulation because of the much denser CG content of the human rDNA promoter (26 CpGs) vs. the mouse rDNA promoter (only 2 CpGs); these potential differences in regulation motivated this study in human cells. We evaluate the functions of rDNA methylation in human cells, which is directly relevant to understanding the regulation of rDNA function in human aging, and to understanding the functional implications of DNA methylation "aging clocks" more generally. We also apply a recently developed technology (dCas9-mediated epigenome editing) to directly test the function of rDNA methylation. Novel findings reported in this study include:
    - Pol I – engaged human rDNA repeats are hypomethylated at sites both in the promoter and the gene body; this contrasts with Pol II transcription, which is coincident with gene body methylation.
    - rDNA copy number remains stable with age in mammals, in striking contrast to findings in other eukaryotes. rDNA copy number instability has been proposed to be a universal feature of the aging genome, and this finding refutes that possibility.
    - Induction of DNA methylation by an average of ~20% along 7-11 of the 26 CpGs in the human rDNA repeat does not measurably inhibit rDNA transcription.
    - Human Pol I and UBTF remain bound to rDNA promoters in the presence of elevated CpG methylation, in contrast to the murine Pol I machinery.

2. Reviewers 1 and 2 questioned our strategy of mapping sequencing data to the consensus ribosomal DNA (rDNA) repeat alone. We followed the approach of Wang & Lemos Genome Research 2019, who initially described the rDNA methylation clock. Wang & Lemos also mapped genomic data to rDNA consensus sequences alone due to the computational efficiency of this approach, and describe a head-to-head comparison of mapping performance outcomes in their Methods section. Importantly, their analysis indicated that the vast majority (>98%) of sequencing reads can be mapped uniquely to the consensus human rDNA repeat (U13369.1). When we launched our study, we also initially compared the performance of mapping to the rDNA repeat consensus sequence alone versus to the whole human genome. We noted very similar performance in both cases, with the possible exception of a modest increase in simple repeat sequences being

erroneously mapped to the intergenic spacer (IGS) region of the rDNA when we mapped to the rDNA repeat alone. As the reviewers pointed out, the IGS contains simple repeat sequences that are also found at numerous other non-rDNA sites in the genome. However, the minor mis-mapping of simple repeats to the IGS did not affect our analyses of non-IGS sequences, which were the focus of this study. We therefore proceeded with mapping to the rDNA consensus sequence only.

3. Reviewers 1 and 2 pointed out that our dCas9-DNMT strategy induced only a 15-20% increase in rDNA methylation and questioned whether we could expect to detect downstream effects in rDNA transcription. While Reviewer 2 suggested that multiple sgRNAs could enhance methylation efficiency, it turns out that this has already been tested for other target genes and shown that multiple sgRNAs cannot increase efficiency of CpG methylation by dCas9-DNMTs (Stepper et al., Nucleic Acids Research 2017). Separately, the goal of this study was to model the effects of age-linked rDNA hypermethylation, which increases by 15-20% over mammalian lifespan (Wang & Lemos 2019; see also our Figure 1). Importantly for interpreting these data, induction of promoter methylation to a similar extent on the mouse rDNA repeat was able to direct detectable repression of rDNA transcription (Santoro et al., 2011). Further, dCas9-DNMT has been previously shown to induce a ~20% increase in CpG methylation of the Pol II target gene *EpCAM* and cause measurable transcriptional repression that was detectable by qPCR (Stepper et al., 2017). In contrast, we were able to induce rDNA methylation to a similar extent and observed no change in the levels of either pre-rRNA or mature rRNA. Because we see that UBF and Pol I remain bound to rDNA in spite of higher CpG methylation (Fig. 7 and Fig. S4), we interpret these data together to indicate that the human Pol I machinery can continue to engage with rDNA in the presence of intermediate levels of CpG methylation.

Reviewer 1

1. “inactivation of rDNA transcription per se does not affect chromatin accessibility, to date only depletion or deletion of UBTF has been found to do this and even this does not enhance CpG methylation, these published findings should be referenced”.

   Our analyses in Figure 2 focus on defining the relationships between chromatin accessibility, transcriptional activity, and CpG methylation throughout the human rDNA repeat. We cannot determine causation from this analysis - meaning whether chromatin accessibility influences CpG methylation or vice versa – and this point is beyond the scope of our study. Our major goal was to test whether induced CpG methylation affects transcription output.

2. The authors overstate their results by writing “actively transcribed rDNA repeats are hypomethylated at their promoter” despite only one SmaI site but many CpG sites exist in the human promoter, the latter having not been assayed.

   We analyzed several pieces of data to come to this conclusion. First, ATAC-Me indicates that ATAC-accessible rDNA repeats are completely devoid of methylation both in their promoter and throughout the gene body; as UBTF binding controls rDNA accessibility (Sanij et al., JCB 2008; Hamdane et al., PLoS Genet 2014), we infer that ATAC-accessible

repeats are engaged with the Pol I transcription machinery and hypomethylated. To more directly probe this question, we evaluated the methylation status of Pol I-bound rDNA repeats at five separate sites by ChIP-chop: two sites in the 5' regulatory region (5' ETS and core promoter, pooled together as "promoter" in Figure 2F) and three sites within the gene body (18S, 5.8S, and 28S, pooled together as "gene body" in Figure 2F). These data clearly indicate that Pol I preferentially binds to these regions when they are hypomethylated, as the extent of CpG methylation at these same sites is higher in input DNA and lower in Pol I-ChIPped DNA. While we do not comprehensively profile CpG methylation status of Pol I-bound DNA, these ChIP-chop analyses are consistent with our interpretation that "actively transcribed (that is, Pol I-engaged) rDNA repeats are hypomethylated at their promoter".

Pol I's preference for binding hypomethylated promoters has been previously described in mouse cells (Santoro & Grummt 2001) and human cells (Brown & Szyf Mol Cell Biol 2007). We confirm this and also report the novel finding that rDNA gene bodies bound by Pol I are hypomethylated. This contrasts with known relationships between Pol II and CpG methylation, where genes actively transcribed by Pol II often have dense gene body CpG methylation.

While we think it is reasonable to infer from ATAC-Me data and ChIP-chop data together that accessible and hypomethylated rDNA repeats reflect transcriptionally active repeats, we appreciate the reviewer's point that we analyzed only a select few CpG sites by Pol I ChIP-chop. We have adjusted the text to make our interpretation more parsimonious (see highlights).

3. The human rDNA promoter contains many CpGs which may not affect transcription when methylated. RRBS and WGBS data can't tell us much if we don't understand which sites, when methylated, affect transcription.

   We agree, and this ambiguity is what motivated us to induce methylation and evaluate the consequences. In plasmid reporter experiments where the human rDNA promoter was fused to a luciferase reporter, it was shown that in vitro methylation of the plasmid potently inhibited transcription in human cells (Ghoshal et al., J Biol Chem 2004). In this study, methylation of 7/26 CpGs was sufficient to induce >75% inhibition of reporter plasmid transcription, while methylation at single sites could induce ~50% inhibition. We neglected to site this relevant study and have included a reference to it in the revised manuscript. Importantly, this plasmid reporter assay does not assess the effects of CpG methylation on the full rDNA repeat in its endogenous genomic context. We were able to induce significant CpG hypermethylation on 11/26 promoter CpGs with one guide (P+G) and on 7/26 CpGs with a second guide (P+A) (Figure 3D). This level of methylation did not induce detectable silencing of rRNA transcription. Instead, we found that both UBF (Fig. 7) and Pol I (Fig. S4) remained bound to rDNA in the presence of CpG hypermethylation.

4. The argument that the mouse rDNA Pol I machinery is "exquisitely sensitive" to CpG methylation is a little misleading as there are only two CpGs in the mouse rDNA promoter. Which of the 26 human CpGs are the critical ones?

   Immediately following this statement in the Discussion, we state that "the human rDNA promoter is significantly more CG-rich than the mouse rDNA promoter". We have revised

this section to emphasize the difference (26 CpGs in human vs. only 2 in the mouse) and discuss this point raised by the reviewer: which are the critical CpGs in the human rDNA? Here again it is relevant to cite the human rDNA promoter reporter assays performed by Ghoshal et al., J Biol Chem 2004. These data indicate that CpG methylation of 7/26 promoter CpGs interferes with transcription from an rDNA reporter plasmid. Notably, it is unclear how generalizable findings from reporter assays are to the genomic context of the endogenous full length rDNA sequence. Our data indicate that partial methylation of 7-11 CpGs in the human rDNA promoter causes no detectable rDNA inhibition, and indeed does not displace UBF or Pol I (Fig. 7; Fig. S4).

5. Antibody SC13125 used for UBF ChIP sees nearly exclusively the shorter transcriptionally inactive UBF2 variant. These data need to be repeated with an antibody that detects both UBF forms.

   We thank the reviewer for raising the important issue of UBTF splice isoforms. Relevant citations demonstrating that the SC13125 antibody recognizes only UBF2 would have been very helpful. The human UBTF *gene is alternatively spliced into full-length UBF1 (exon 8 retained) and UBF2 (exon 8 spliced out). The deletion of exon 8 results in a 37 amino acid deletion in UBF2 corresponding to residues 221-268 in HMG box 2 of UBF1 (see Ensembl entry ENSG00000108312.16). The truncation of HMG box 2 makes UBF2 a far less potent transcriptional activator than UBF1. Because of the small molecular weight difference between these two isoforms, preference of an antibody for one vs. another isoform is not readily apparent by Western blotting. However, according to the manufacturer of the UBTF antibody used in this study, the immunogen for the SC13125 antibody corresponds to residues 1-220 of UBTF1, which is immediately N-terminal to the residues deleted in UBF2 (AAs 221-268, encoded by exon 8). The antibody's immunogen is thus entirely sequence that is shared between UBF1 and UBF2. Further, a previous study performed immunoprecipitation followed by mass spectrometry using this antibody and reported detection of UBF1-specific peptides (Drakas et al., PNAS 2004). Therefore, absent our knowledge of any evidence to the contrary, we conclude that this antibody recognizes UBF1 and possibly also UBF2.*

   We thank the reviewer for raising this point and have adjusted the text to avoid the misleading implication that we are unambiguously detecting only the UBF1 isoform; all mentions of "UBF1" in the revised text have been replaced with "UBTF".

   Setting aside the question about the UBTF antibody reagent used, we observe consistent results by evaluating both UBTF (Figure 7) and Pol I (Figure S4) binding to rDNA in spite of CpG methylation; therefore, we conclude that the human Pol I machinery is not displaced from the human rDNA promoter by intermediate levels of CpG methylation.

Reviewer 2

1. There is very little discussion concerning the methylation status of the IGS…the Kobayashi lab has convincingly demonstrated that rDNA repeats fall into 2 classes. Those in which the supposedly active repeats lack methylation on promoters and coding regions and those in which both promoters and coding regions are heavily methylated. In both cases the IGS is fully methylated.

We cite this study in the Discussion (reference 18 in bibliography) and agree that this work is relevant to ours; we have adjusted the text to emphasize this point. Notably, this previous analysis of CpG methylation patterns by long-read sequencing implied that active repeats may be entirely hypomethylated along their coding sequence; our data more directly demonstrate this both by ATAC-Me and by Pol I ChIP-chop (Fig. 2).

2. There is no description of how rRNA levels were assessed. I suggest this could be further complemented by in vivo incorporation studies such as EU labeling.

   We apologize for this lack of clarity. rRNA levels were assessed by qPCR of the 45S pre-rRNA (Fig. 3A) and of mature 28S rRNA (Fig. 3B), and these data are presented as a fold change in each rDNA-targeting sgRNA compared to a non-targeting control sgRNA. The primersets used are listed in Supplementary Table 1.
   While we agree that EU labeling could be useful for detecting nucleolar transcription, qPCR detection of the 45S rRNA also sensitively reports nascent transcription and we think is sufficient to address this question.

Reviewer 3

1. The study points to differences between mouse and human rDNA and the effect of DNA methylation on transcriptional output. Did the mouse rDNA dataset also measure transcription output to correlate with DNA methylation age differences?

   The original study that defined the rDNA methylation clock (Wang & Lemos Genome Research 2019) did not evaluate rDNA transcription in parallel. More generally, the relationship of age-linked "clock" CpG methylation sites to expression / function of CpG methylated loci is very unclear, and testing the potential relationship between age-linked rDNA methylation and function was the major goal of this study.

2. Did the spacer promoter also get methylated and did that affect UBF and Pol I binding?

   While the existence and function of a spacer promoter has been more clearly defined in the mouse rDNA repeat, recent evidence indicates that the Pol I transcription machinery also binds a second location about 800 bp upstream of the core promoter in the human rDNA repeat (Mars et al G3 2018). The guides that we used to direct CpG methylation recognize single unique sites in the core rDNA promoter and do not recognize sequences in this putative spacer promoter, and we did not analyze methylation at the spacer promoter. Analysis of the spacer promoter is generally beyond the scope of this study, as it is unknown whether there is any relationship between spacer promoter methylation and aging progression.
