# Supplementary material for: Modeling the consequences of age-linked rDNA hypermethylation with dCas9-directed DNA methylation in human cells
Source: PLoS One. 2024 Dec 12;19(12):e0310626. doi: 10.1371/journal.pone.0310626 (PMC11637357; doi:10.1371/journal.pone.0310626)
Supplement: Attachment — Submitted filename: Blokhina & Buchwalter response to reviewers 8-7-24.docx [file pone.0310626.s008.docx]

**Response to Reviewers – Blokhina & Buchwalter – PONE-D-24-23946**

We thank the reviewers and editorial staff for dedicating the time to re-review our manuscript. Our responses to each reviewer’s comment follow below.

R1.1: “I am of the opinion that the activity levels of the rDNA are far more important to aging than its methylation levels, and feel that the (original) response to Reviewer 1.1 comment simply attempts to dodge this key issue.”

We understand the reviewer’s point and agree that rDNA activity levels are clearly functionally important. We apologize that our original response to this point did not satisfactorily address the reviewer’s critique. The overarching goal of our study was to test how, if at all, age-linked rDNA CpG hypermethylation affects the function of human rDNA loci. It is exactly because of the point the reviewer makes – that activity levels of rDNA are what are most functionally important – that we set out to determine whether hypermethylation of human rDNA loci would have any measurable effect on rDNA activity. More generally, the relationship of age-linked “clock” CpG methylation sites to expression / function of CpG methylated loci is very unclear, and testing the potential relationship between age-linked rDNA methylation and function was the major goal of this study. Our finding that the human rDNA locus can tolerate hypermethylation to a similar extent as that observed during the aging process without any detectable changes to activity is relevant both to our understanding of human rDNA regulation and to our interpretation of DNA methylation clocks.

R1.2: “The comment… concerning the UBTF antibody again attempts to sidestep the very important issue of specificity.”

We thank the reviewer for providing an important and relevant reference (Theophanous et al., JBC 2023) which, as the reviewer describes, shows using RNAi that the Santa Cruz UBF antibody preferentially recognizes the UBF2 isoform over the UBF1 isoform. We have now cited this study in our discussion of the relevant experiments (Figure 7) in the Results section. Importantly, as the reviewer notes, UBF1 and UBF2 heterodimerize and thus immunoprecipitation of either isoform is likely to enrich some UBF1, the isoform that is capable of rDNA promoter TF activity. Indeed, we detect robust and specific UBF binding at the human rDNA promoter but not at other loci – such as the 5S rDNA locus - in our ChIP assays. Importantly, our interpretation that the human rDNA transcription machinery remains bound to rDNA in spite of CpG methylation is supported both by UBF ChIP (Fig. 7) and by Pol I ChIP (S4 Fig.).

R2.1: “It was unclear whether the authors have tried to align the reads to the Genome assembly T2T-CHM13v2.0 to study transcription and DNA methylation of different loci?”

We did not attempt to align sequencing reads to the Telomere to Telomere genome assembly. We agree that the T2T assembly provides exciting future opportunities for understanding the structure and regulation of rDNA repeat arrays, and we also cite recent work from the Kobayashi laboratory that used long-read sequencing approaches to resolve rDNA arrays. We now state in the Discussion that the T2T assembly will enable future dissections of rDNA repeats in their distinct genomic context.

R2.2: “A ChIP of RNA pol I or RRN3 would be instrumental in assessing the level of polymerase binding at the promoter. Figure S4C shows a Pol I ChIP – is it to understand that no change in Pol I level occurred?”

Yes, the reviewer is correct; we performed Pol I ChIP analyses in the absence or presence of dCas9-mediated hypermethylation and found that Pol I binding to the rDNA locus was unchanged. As we note in the Discussion, however, this average measurement of Pol I binding to rDNA promoters cannot rule out the “possibility that transcription is further accelerated on remaining hypomethylated rDNA repeats to…sustain rRNA synthesis. Nevertheless, our data indicate that human UBF and Pol I can tolerate significant levels of rDNA promoter methylation and remain bound to methylated rDNA repeats.”

R2.3: “Is level of TTF1 at the promoter changed?”

We thank the reviewer for this insightful question. We did not evaluate the binding of TTF1 to rDNA. Interestingly, work from the Moss laboratory indicates that TTF1 is the sole element of the rDNA transcriptional machinery that remains bound to the rDNA when UBF is acutely ablated (Hamdane et al., PLoS Genet 2014). We speculate, then, that TTF1 would remain bound to rDNA even if UBF and/or Pol I were displaced by DNA methylation.

R2.4: “It would also be interesting to see the transcription from the upstream promoter…does the higher DNA methylation at the promoter affect transcription upstream of the promoter?”

The existence and function of a spacer promoter has been more clearly defined in the mouse rDNA repeat and has been little studied in the human rDNA repeat. Recent evidence indicates that the human Pol I transcription machinery binds a second location about 800 bp upstream of the core promoter in the human rDNA repeat (Mars et al G3 2018). This second locus does contain UBF1 binding sites and a core promoter motif, suggestive of its function as a spacer promoter. The guides that we used to direct CpG methylation recognize single unique sites in the core rDNA promoter that are not homologous to this putative spacer promoter, and we did not analyze methylation at the spacer promoter.

To address the reviewer’s question of whether induced methylation at the core promoter could affect upstream transcription from the spacer promoter, we designed two primersets to amplicons just downstream of the putative transcription start site of the human spacer promoter described by Mars et al. and diagrammed in Figure 5 of that study. These primersets are:

Set 1: ~80 bp product immediately downstream of putative spacer promoter TSS

Fwd: TGG GGG AGA GGC TGT CG

Rev: CTA CGG CGC GCT GGT C

Set 2: ~100 bp product ~100 bp downstream of putative spacer promoter TSS

Fwd: TAG CTC CCG AGG CCC GA

Rev: GGC CAG GAT GAG CGG AC

We used these two primersets to evaluate rRNA abundance in HEK293T cells after induction of dCas9-DNMT and a non-targeting control (NTC) sgRNA vs. induction of dCas9-DNMT and our “P+A” core promoter-targeting sgRNA. (See S2 Fig for diagram of guide position.) Both of these primersets did amplify RNA, but did not detect any significant difference in RNA abundance in cells treated with the promoter-targeting guide compared to those treated with the non-targeting guide. (For reference, this guide induced >20% increase in promoter CpG methylation in HEK293Ts; see Fig. 4C). Overall, this outcome is consistent with the lack of effect on rRNA abundance that we report in Figure 5.

We thank the reviewer for raising this question and share the result of this experiment here; however, because so little is known about transcripts originating from the putative human spacer promoter, we feel that this analysis is too preliminary to be included in the manuscript.

R2.5: “In Supplementary Figure 1 - the variation in the ChIP experiment is high - is the highest binding an outlier?”

The data shown in S1 Fig are only intended to demonstrate the ability of primersets used to detect Pol I binding along the rDNA; while one dataset may be an outlier this does not affect our assessment that these primersets can effectively detect Pol I binding above IgG background along the rDNA repeat. These same primersets are used in Figure 2 in independent ChIP-Chop experiments.
